# Automatic classification of sleep stages using EEG signals and convolutional neural networks

**DOI:** 10.1371/journal.pone.0297582

**Published:** 2024-01-26

**Authors:** Ihssan S. Masad, Amin Alqudah, Shoroq Qazan

**Affiliations:** 1 Department of Biomedical Systems and Informatics Engineering, Yarmouk University, Irbid, Jordan; 2 Department of Computer Engineering, Yarmouk University, Irbid, Jordan; XJTLU: Xi’an Jiaotong-Liverpool University, CHINA

## Abstract

Sleep stages classification is one of the new topics in studying human life quality because it plays a crucial role in getting a healthy lifestyle. Abnormal changes or absence of normal sleep may lead to different diseases such as heart-related diseases, diabetes, and obesity. In general, sleep staging analysis can be performed using electroencephalography (EEG) signals. This study proposes a convolutional neural network (CNN) based methodology for sleep stage classification using EEG signals taken by six channels and transformed into time-frequency analysis images. The proposed methodology consists of three major steps: (i) segment the EEG signal into epochs with 30 seconds in length, (ii) convert epochs into 2D representation using time-frequency analysis, and (iii) feed the 2D time-frequency analysis to the 2D CNN. The results showed that the proposed methodology is robust and achieved a very high accuracy of 99.39% for channel C4-A1. All other channels have accuracy values above 98.5%, which indicates that any channel can be used for sleep stage classification with high accuracy. The proposed methodology outperformed the methods in the literature in terms of overall accuracy or single channel accuracy. It is expected to provide a great benefit for physicians, especially neurologists; by providing them with a new powerful tool to support the clinical diagnosis of sleep-related diseases.

## Introduction

Sleep disorders are common among the world population, leading to serious health problems that affect the quality of life. Insomnia, parasomnias, sleep-related breathing and movement disorders, hypersomnias, narcolepsy, and circadian rhythm disorders are some of the sleep-related diseases [1]. In order to detect and classify the sleep stages, several techniques can be used such as Polysomnogram (PSG) which is a set of multivariate signals recorded from subjects under study and collected during an entire night of sleep. The PSG consists of different signal recordings such as the electrocardiogram (ECG), electrooculogram (EOG), electroencephalogram (EEG), and electromyogram (EMG) [2].

Using these PSG signals, sleep can be classified into different stages: wake (W), three non-rapid eye movement (NREM) stages (N1-N3), and rapid eye movement (REM). The process is usually done manually by sleep experts who classify sleep stages by visually inspecting and evaluating the PSG signals for a specific time frame called epochs, then determining the class based on different criteria. The classification is based on a guideline developed by the American Academy of Sleep Medicine (AASM) [3].

Sleep stages or scores generally consist of two major types, REM sleep and NREM sleep (Consisting of three different stages). Each stage affects specific brain waves besides neural activity. During sleep, the person makes several cycles among all stages of NREM and REM sleep during the night, with increasingly longer, deeper REM periods occurring toward morning. The following paragraphs describe the sleep stages [4]:

1. The first stage of sleep, which is a part of the NREM sleep stage, is the main changeover from wakefulness to sleep, and usually lasts for several minutes of relatively light sleep. During this stage, heartbeat decreases, while breathing and eye movements slow down. Moreover, brain waves begin to change and slow their pattern compared to daytime wakefulness [5].
2. The second sleep stage is a part of NREM sleep as well; it is a period of light sleep that makes heartbeat and breathing slower and body muscles more relaxed. Besides, brain waves activity will slow down, and the person spends more time in this stage than he/she does in other stages [6, 7].
3. The third sleep stage is the final stage of NREM sleep. The heartbeat, breathing, and brain waves will be at their lowest levels during this stage, and all muscles will be relaxed [6].
4. The final stage is the REM sleep stage in which the brain wave pattern becomes closer to the pattern in wakefulness, and heart rate becomes higher, while breathing rate becomes faster and irregular [7, 8].

The EEG signal describes the brain’s electrical activity. The nature of this signal is non-linear and nonstationary, so it is challenging to deal with it and get information about brain state directly in the time domain. The EEG signals are recorded from electrodes placed on the scalp with amplitude of about 10 μV to 100 μV and a frequency in the range of 1 Hz to about 100 Hz [9]. In this study, the standard ear loop reference electrode (A1 and A2) has been used with the following midline channels: Central channels (C1 and C2), Occipital channels (O1 and O2), and Frontal channels (F3 and F4); which are the standard channels used by the AASM to record sleep-related EEG signals [10].

The visual inspection and manual evaluation of the PSG signals are time-consuming, complex and costly processes requiring expert technicians. On the other hand, EEG is the most robust signal used for sleep stage classification, yet, it is very hard to visually detect sleep stages by only inspecting variations because of their random appearance and chaotic nature [11]. In order to successfully classify the sleep stages using EEG signals and to objectively make the proper diagnosis and decision, automated detection and classification systems must be developed to help neurologists and sleep experts in such complex tasks. In addition, advanced signal processing techniques and artificial intelligence algorithms must be applied to make sleep stage classification simpler and more robust [4]. The goals of this study are to build and validate a new fast and accurate sleep stage classification methodology by designing a new convolutional neural network (CNN) model; in addition to find the best EEG channel(s) that can be used to classify sleep stages efficiently.

## Literature review

In recent years, researchers have paid attention to sleep stage classification using different signals, especially EEG signals. Many approaches have been proposed for EEG pre-processing including dividing signals into epochs and classification of the epochs. These studies have developed different methods to classify sleep stages using EEG signals accurately. The following literature review is categorized based on the used methodology: machine learning, Long-Short Term Memory (LSTM), 1D-CNN, and finally 2D-CNN models.

### A. Machine learning-based methods

Khalighi et al. [2] proposed an automatic methodology for two scenarios of sleep stage classification. The first scenario was to classify into sleep or awake, then classify sleep into N1, N2, N3, and REM. The second scenario was to directly classify EEG into multiclass stages (N1, N2, N3, REM, and awake). They used a combination of time, frequency, and time-frequency features that were extracted using the maximum overlap wavelet transform (MODWT) and shift-invariant transform. Then the extracted features were fed into two-step features selection to select the discriminative features, which are classified using support vector machines (SVMs). The authors reported that the best performance achieved for the first scenario was using 6 channels with an accuracy of 94.58%, while for the second scenario was using 9 channels with an accuracy of 92.04%. On the other hand, a decision tree using a multiclass SVM for multiclass sleep stage classification was proposed by Lajnef et al. [7]. The method was based on obtaining a decision tree using the hierarchical clustering technique, the tree was fed with several extracted time domain and frequency domain features. Their proposed classifier has been evaluated using k-fold cross-validation, which resulted in an overall accuracy of 92%.

Gupta et al. [12] proposed a time-frequency representation (TFR) method depending on the Fourier-Bessel decomposition method (FBDM) to decompose the non-stationary signal into a finite number of Fourier-Bessel intrinsic bands functions (FBIBFs). To get a fixed number of FBIBFs, they have suggested a zero-phase filter-bank-based (FBDM). The created FBDM was utilized to classify six distinct sleep stages using the CNN classifier to classify TFR images. Their proposed model achieved an accuracy of 91.90%. Grieger et. al. [13], on the other hand, proposed a classification system in mice that evaluated the classical sleep stages of Wake, REM, NREM and pre-REM sleep stages using a simple neural network design. The performance obtained 0.95 F1 score if the network was restricted with an out-of-sample; while in case of unrestricted networks, they obtained a performance of 0.5 F1 score.

Using a machine learning-based system to classify sleep stages, Satapathy et. al. [14] extracted 12 statistical features from each signal, then three different combinations of these features have been used. The ISRUC‐Sleep database features were tested with two categories (sleep disorder and healthy), and fed to three different classifiers including Decision Tree (DT), K-Nearest Neighbor (KNN), and Random Forest (RF). The results showed that all classifiers achieved an accuracy higher than 90%, while the RF had the highest accuracy of 96.7%. In addition, Surantha et. al. [15] reported the use of the Heart Rate Variability (HRV) feature, which was calculated from ECG signals. They used a combination of extreme learning machine (ELM) and particle swarm optimization (PSO) for features reduction and selecting the best-hidden nodes numbers. The results showed accuracies ranging between 62.66% and 82.1% for different number of classes.

### B. LSTM-Based methods

Ghimatgar et al. [16] combined deep learning and hidden Markov models (HMM) using multichannel EEG to improve the accuracy of sleep stage classification for neonates. The features were extracted from 30-second EEG segments and were reduced using Modified Graph Clustering Ant Colony Optimization (MGCACO) algorithm. They utilized a bi-directional LSTM (BiLSTM) network as a sleep stage classifier where the number of channels was optimized using a sequential forward selection algorithm. The authors used HMM as a post-processing technique to reduce the number of false positives. The model has been validated using two methods: the leave-one-out cross-validation technique, resulting in an overall accuracy of 82.4%, and k-fold cross-validation with an overall accuracy of 78.9%. Choi et al. [17] achieved an accuracy of 73.9% with a PSG-based system for sleep stage classification using Polyvinylidene Fluoride Film Sensor (PVDF). The PVDF sensor was used for long-term, unconstrained, and stable physiological signal recording and monitoring. Then the LSTM model was used to classify the recorded signal into four sleep stages; the LSTM consisted of three layers: two BiLSTM and one fully connected layer. However, when Ziliang et al. [18] used LSTM and a 30-second-time point time-frequency spectrum as input, the accuracy was 87.4%. Moreover, Michielli et al. [5] proposed another deep learning methodology using a cascaded LSTM recurrent neural network (RNN) for sleep stage classification where a single-channel EEG signal features was used as an input for the cascaded model. Their results showed that the LSTM-RNN cascade model scored an accuracy of 90.8% and 83.6% for LSTM and RNN, respectively.

### C. 1D CNN-Based methods

Satapathy et al. [19] achieved 97.22% accuracy using deep learning methods to detect multiple sleep diseases using three different types of signals as inputs. While Zhu et al. [20] achieved 93.7% with an attention-based CNN model that used the PSG signal as input, Loh et al. [21] proposed a 1D-CNN based on a deep learning model to classify sleep stages. The model was proposed for cyclic alternating pattern (CAP) detection and homogenous 3-class sleep stage classification (W, REM, and NREM sleep) using EEG recordings. The result for the 3-class sleep stage classification of the proposed CNN network achieved an accuracy of 90.46%, however, it showed a poor accuracy of 73.64% and 52.99% using balanced and unbalanced CAP datasets, respectively. As well, Yildirim et. al. [22] proposed a 1-D CNN model using EEG and EOG signals where the accuracy ranged between 91% and 98% depending on the number of classes.

### D. 2D CNN-Based methods

A joint classification and prediction CNN framework has been proposed by Phan et al. [23] for sleep stage classification. The framework was based on a simple, efficient, and powerful multi-task CNN model that was used for automated sleep stage classification using single epoch signals as input. The framework used ensemble features extracted from three signals (EMG, EEG, and EOG), then these features were converted to a time-frequency image that was used as input to the CNN. The framework has been tested using two publicly available datasets; the first one was sleep-EDF which achieved an accuracy of 82.3%, and the Montreal Archive of Sleep Studies (MASS) with an accuracy of 83.6%.

Jadhav et al. [6] proposed automated classification of sleep stages using deep learning methods. They used single-channel EEG to generate a time-frequency spectrum of EEG signals; they used continuous wavelet transform (CWT) to extract the RBG image, which was fed to the pre-trained Squeezenet CNN model. The authors achieved an accuracy of 83.34%, 83.61%, and 83.17% for 30-second epoch for Morse, Bump, and Amor CWT, respectively. Moreover, Moradi et al. [8] used transferee learning for sleep stage classification using pre-trained CNN models using the Sleep Heart Health Study (SHHS) PSG dataset. The authors used two signals as input to Wanger-Ville Distribution (WVD) to convert it into 2D input (image) for the AlexNet CNN model. The two signals were ECG and Photoplethysmography (PPG). The proposed method achieved an accuracy of 95.25% using ECG and 94.63% using PPG.

Cui et al. [24] proposed CNN using fine-grained signal segments for sleep stage classification. The methodology was based on fine segmentation with a length of 30 seconds of 11 signals that were reshaped as a time series together as input for 8-layer CNN model. The model has been successfully applied on the ISRUC-Sleep public dataset and can classify 5 classes of sleep stages with accuracies of 90%, 86%, 93%, 97%, and 90% for stage W, stage N1, stage N2, stage N3, and stage REM, respectively.

Yuan et al. [25] proposed an end-to-end hybrid deep learning network using a self-attentive method for multivariate sleep stage classification. This model detected correlation over time and extracted deep features from heterogeneous biomedical signals fed to it. The authors used a channel-wise attention-based model to integrate information from multi-views to enhance the extracted deep features. The method was tested using the UCD PSG dataset, provided by St. Vincents University Hospital and University College Dublin, and achieved an accuracy of 73.28%.

Eldele et al. [26] proposed automated classification of sleep stages using attention-based deep learning architecture called AttnSleep. This method used single-channel EEG signals to extract features by two modules: the first module was based on a multi-resolution convolutional neural network (MRCNN), which extracts low and high-frequency features. The second model was adaptive feature recalibration model (AFR) to improve the quality of the extracted features. These modules used a temporal context encoder (TCE). This technique impacts multi-head attention to ensure the capture of the temporal dependencies among the extracted features. The results indicated the stability of the method with an accuracy above 84%. Table 1 below shows a summary of the reviewed literature.

**Table 1:** Summary of all survived literature.

Ref #	Methodology	Accuracy %	
[2]	Time, frequency, and time-frequency features with SVM	94.58 92.04	
[26]	1D-CNN	97.22	
[10]	Cascaded LSTM and RNN	86.7	
[23]	Combined BiLSTM with HMM	CV	82.4
		K-Fold	78.9
[30]	2D-CNN with time-frequency image with two datasets	82.3 83.6	
[27]	Attention-based 1D-CNN with two datasets	93.7 82.8	
[31]	2D CNN model	91.2	
[13]	AlexNet model with Wanger-Ville Distribution	ECG	95.25
		PPG	94.63
[32]	Self-attentive hybrid deep learning	73.28	
[24]	LSTM	73.9	
[12]	Time-domain and frequency domain features with SVM	92	
[11]	Squeezenet model with the continuous wavelet transform with 30 seconds	Morse	83.34
		Bump	83.61
		Amor	83.17
[25]	LSTM	87.4	
	CNN	84.4	
[33]	Multi-resolution convolutional neural network with temporal context encoder	>84	
[28]	1D-CNN with cyclic alternating pattern	90.46	
[19]	Time-frequency representation with Fourier-Bessel decomposition	91.90	
[20]	Simple neural network	restricted	0.5F1
		unrestricted	0.95F1
[21]	Random forest	96.7	
[22]	Extreme learning machine with particle swarm optimization	6 classes	62.66
		4 classes	71.52
		3 classes	76.77
		2 classes	82.1
[29]	1D-CNN	2 classes	98.06
		3 classes	94.64
		4 classes	92.36
		5 classes	91.22
		6 classes	91.00

## Materials and methods

**Fig 1:**
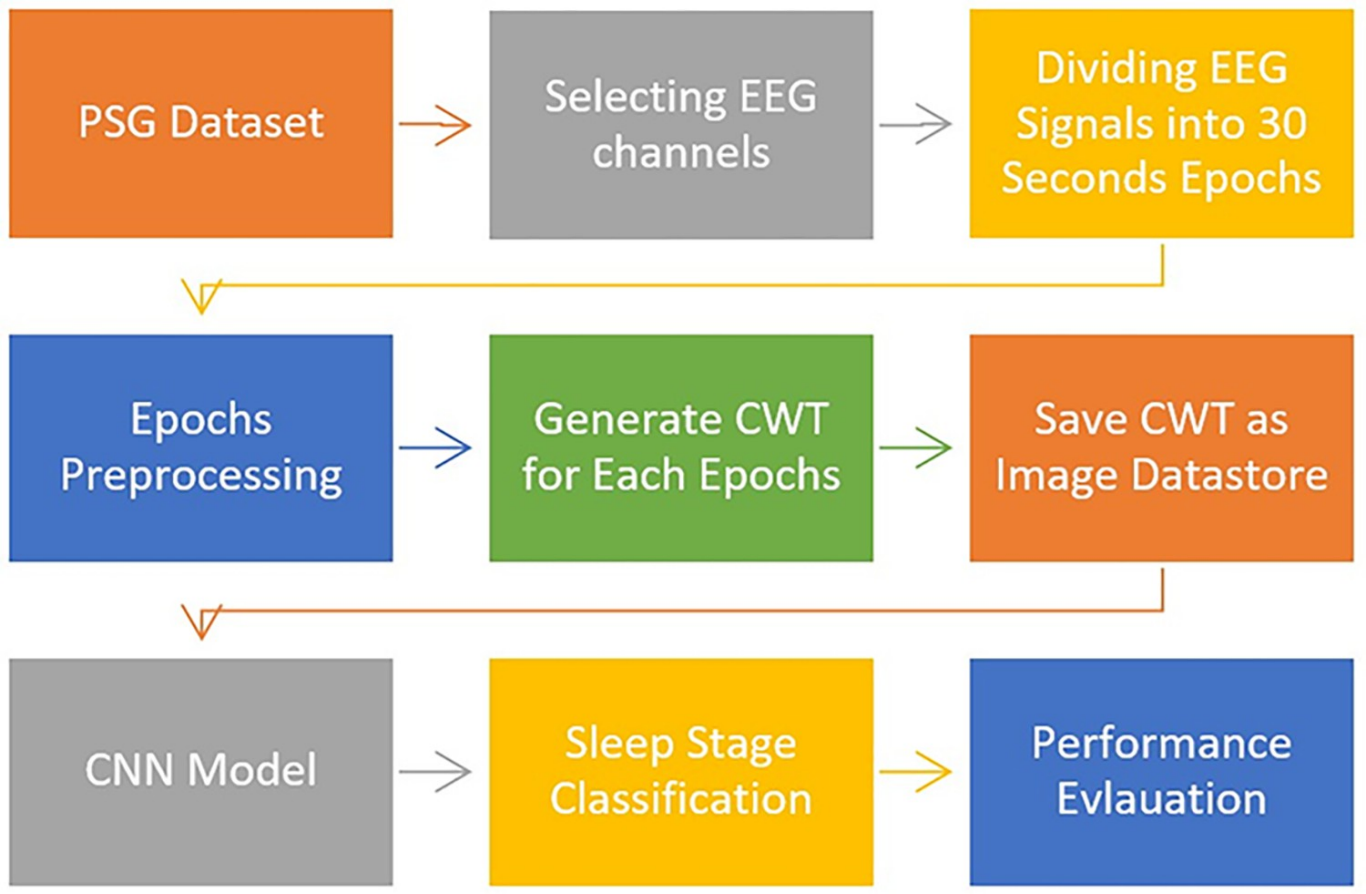
Block diagram of the proposed methodology.

The proposed methodology for sleep stages classification is illustrated in the block diagram shown in Fig 1.

### Participants

The ISRUC-Sleep dataset recorded at the Sleep Medicine Centre of the Hospital of Coimbra University, Portugal, in the period between 2009 and 2013 has been utilized [10]. This dataset is one of the largest publicly available datasets such that it can be used to make a comprehensive study about the sleep stage classification using different EEG channels. The data used in this study contained 100 records of all PSG signals from 100 different subjects (45 female and 55 male) with different conditions, either healthy or patients with sleep disorders. All subject’s data were recorded using one data acquisition device per session [10]. In this dataset, each subject’s record was labeled based on visual inspection of two sleep experts based on the AASM standard [13, 17].

### EEG signals preprocessing

**Fig 2:**
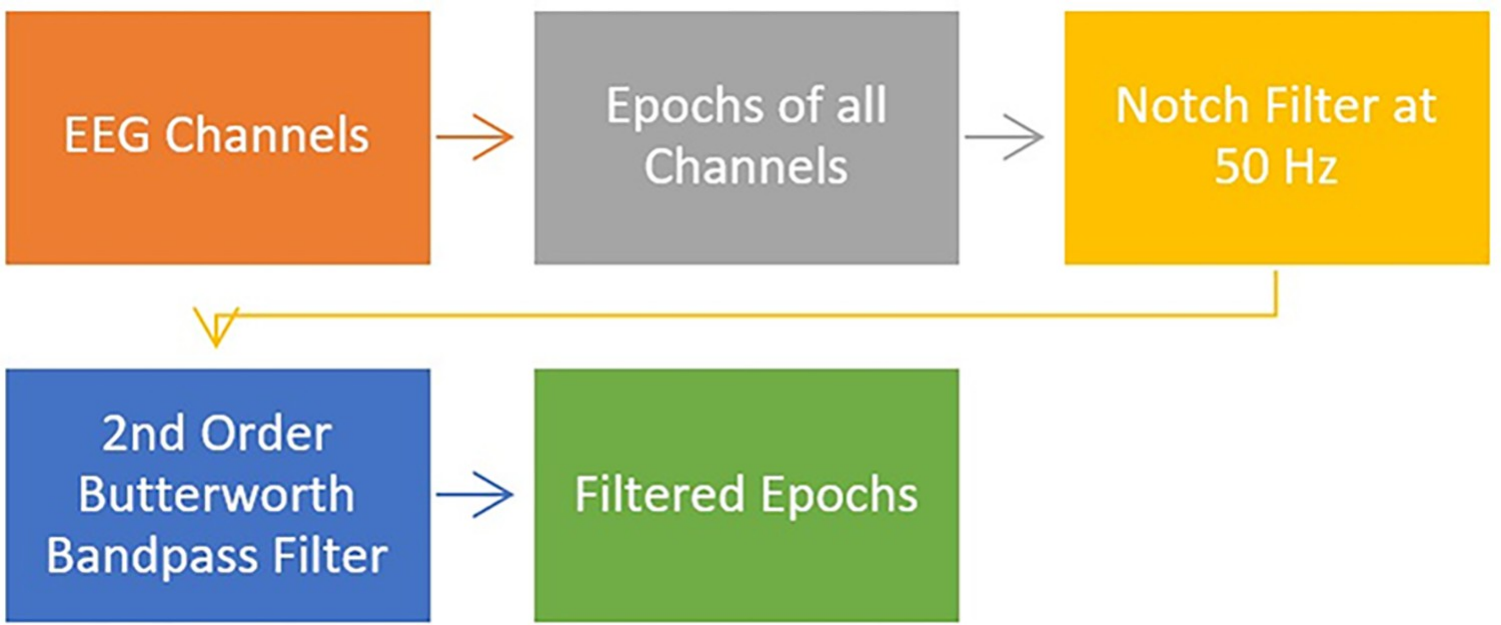
Block diagram of the preprocessing techniques.

Because EEG signals from the ISRUC-SLEEP Dataset were characterized with a low signal-to-noise ratio (SNR), the preprocessing phase was applied to enhance the quality of the signals; for example, recorded signals of EEG channels were filtered to remove noise and undesired background noise and artifacts [10]. In this study, the filtering phase consisted of two main stages: (1) a notch filter to eliminate the 50 Hz powerline interference; (2) a 2^nd^ order bandpass Butterworth filter with a lower cutoff frequency of 0.3 Hz and a higher cutoff frequency of 30 Hz [4, 10]. Fig 2 shows the block diagram of the preprocessing techniques.

### Continuous Wavelet Transform (CWT)

The EEG signals are composed of different frequency bands, and it is hard to determine them only using frequency analysis (Fourier Transform). Usually, in EEG signal classification problems, the main aim is to localize the time at which each frequency occurs. So, the EEG signals have been transformed into the time-frequency domain to represent each frequency occurrence time [27, 28]. CWT is the most commonly used and powerful time-frequency analysis tool in which a certain family of wavelet functions used to decompose a signal in the time-frequency domain instead of a sinusoidal signal like Short Time Fourier Transform (STFT) [29, 30]. In contrary to STFT, The CWT doesn’t use a fixed resolution over all windows. The time resolution and frequency resolution in the high and low frequencies can be altered by adjusting the scale and translation parameters [31, 32]. Moreover, given a signal *x*(*t*), the CWT can be defined as [31, 32]:

$$C_{a}(b)=\frac{1}{\sqrt{a}}\int_{-\infty }^{\infty }x(t)\varphi (\frac{t-b}{a})dt$$ (1)

Where *a* is a scale parameter, *b* is a translation parameter, and *φ*(*t*) is the mother wavelet function known as the mother wavelet.

The scale can be converted to frequency by [31, 32]:

$$F=\frac{F_{c}\times F_{s}}{a}$$ (2)

Where *F*_*c*_ is the center frequency of the mother wavelet, *F*_*s*_ is the sampling frequency of signal *x*(*t*), and *a* is a scale parameter.

**Fig 3:**
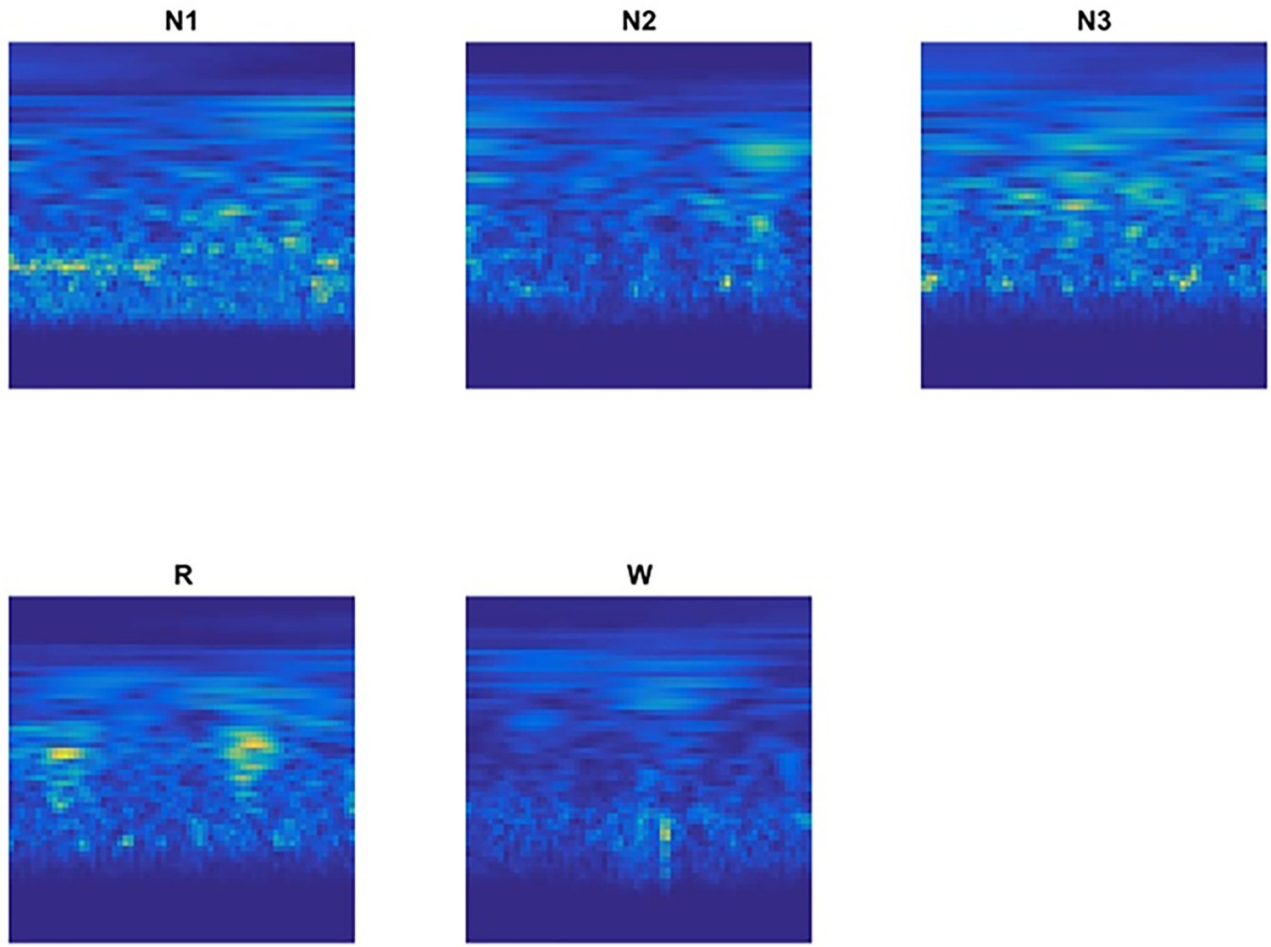
CWT during different sleep stage of C4-A1 EEG channel signal; N1: Sleep Stage 1; N2: Sleep Stage 2; N3: Sleep Stage 3; R: REM; and W: Wake.

Among all available wavelets, the choice of mother wavelet is critical because it directly affects the time-frequency analysis. The bump wavelet has been utilized to analyze EEG signals because it has a narrower frequency variance as the mother wavelet. Fig 3 shows a sample STFT for EEG signal (F3-A1) during different sleep stages. This Figure shows a significant difference among different epochs, especially in the high frequency ranges.

### Proposed CNN model

**Fig 4:**
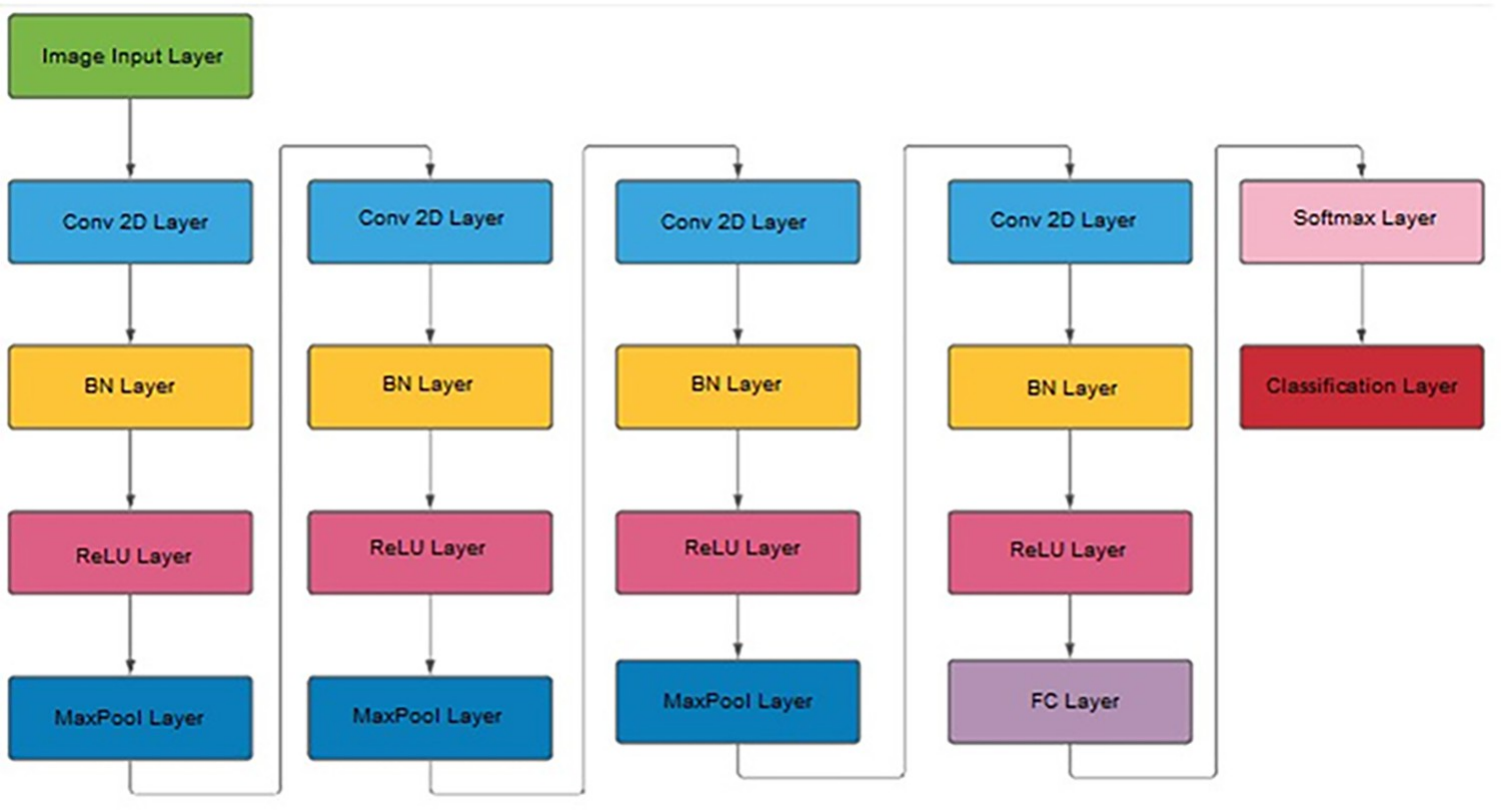
The proposed 19 layers CNN model.

A 19-layer CNN architecture was developed (Fig 4) to classify and differentiate input EEG epochs into 5 classes. Compared to similar pretrained networks commonly used with transfer learning techniques, the proposed model architecture will reduce the number of layers; namely, Densnet has 201 layers, ResNet has 101 layers, and GoogleNet has 144 layers. Reducing the number of slices will decrease the time required for training and finding probabilities from newly input CWT images and reduce the computing resources required to run the system. Table 2 shows the details about layers in the proposed CNN model architecture.

**Table 2:** Values for information of the layers in the proposed CNN Architecture.

#	Layer	Info	Value	#	Layer	Info	Value
1	Input Layer	Size	64×64×3	10	Conv_3	Filters	32
2	Conv_1	Filters	128			Kernel Size	3×3
		Kernel Size	7×7			Activation	ReLU
		Activation	ReLU	11	Batch_Norm_3	Channels	32
3	Batch_Norm_1	Channels	128	12	ReLU_3	-	-
4	ReLU_1	-	-	13	Maxpol_2	Kernel Size	2×2
5	Maxpol_1	Kernel Size	3×3			Stride	2×2
		Stride	2×2	14	Conv_4	Filters	16
6	Conv_2	Filters	64			Kernel Size	3×3
		Kernel Size	5×5			Activation	ReLU
		Activation	ReLU	15	Batch_Norm_4	Channels	16
7	Batch_Norm_2	Channels	64	16	ReLU_4	-	-
8	ReLU_2	-	-	17	FC Layer	Size	5
9	Maxpol_2	Kernel Size	3×3	18	Softmax Layer	-	-
		Stride	2×2	19	Classification Layer	-	-

Using Fig 4 and Table 2, it is worth noting that the proposed model has 4 blocks for extracting very deep features from epochs CWT. These blocks form the core of the model to extract both deep and general features and obtain the most discriminative ones. The uniqueness of the proposed model lies in the combination of the deep features extracted from the four consequent convolution layers separated by Rectified Linear Unit (ReLU) and the batch normalization layer. General features were extracted using the x-box technique which allows using both general and minor changes in the CWT images. Furthermore, the proposed model will improve the flow of information and gradients through the network, making the optimization of very deep networks tractable. Other advantages of the model include strong features propagation, feature reuse and combination, and substantial reduction in the number of parameters. The network weights and biases were initialized using “glorot” weight initialization, where a small gaussian value with a zero mean is initially assigned to each weight. Finally, the network will be trained end-to-end.

The confusion matrix has been used in order to test the effectiveness of the proposed model by comparing the device output to the reference labels or data [33, 34]. Common metrics of model performance (i.e. such as accuracy, specificities, sensitivity, and precision as well as F1-Score can be extracted from the confusion matrix [35–37].

## Results

The epochs of all EEG channels from patients were stacked together resulted in a total of 87,001 epochs (Table 3 shows the total number of samples per class), while the labels were scored based on the PSG technicians’ reports. The CWT images dataset for each EEG channel epoch was then generated and saved to its corresponding label folder. The bump wavelet has been used to detect the narrower variance in frequency with sampling frequency (Fs) equal to 200. Each folder of EEG channel epochs CWT images was divided into three sub-datasets, namely, training, validation, and testing with the following ratios 70%, 15%, and 15%, respectively. The model trained using Adam optimizer with an initial learning rate of 0.001, mini-batch size of 128, max epochs of 100, validation frequency of 100, and using parallel processing technique. For each channel, the training and validation accuracy and loss curves were evaluated, in addition to the testing confusion matrix.

**Table 3:** Number of Samples per class.

Stage	Number of Samples
W	19,840
N1	11,113
N2	27,427
N3	17,340
REM	11,281
**Total**	**87,001**

**Fig 5:**
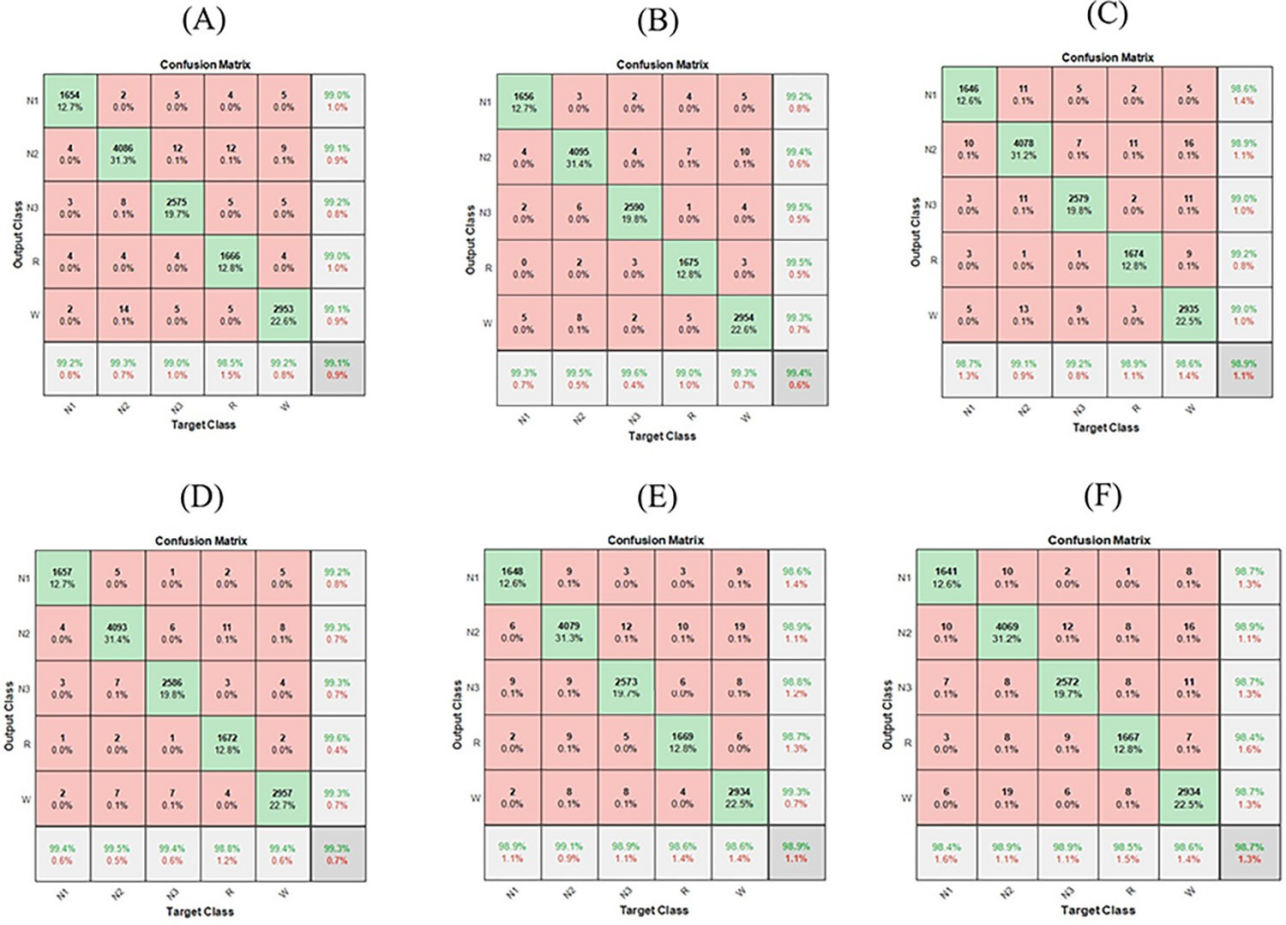
Testing Confusion Matrix for Channel Epochs: A) C3-A1, B) C4-A2, C) F3-A1, D) F4-A2, E) O1-A2, F) O2-A1.

All CWT images generated from EEG channels epochs have been fed to the proposed CNN model architecture. Then, an independent test dataset was used in order to evaluate the performance of CNN model architecture in classifying the sleep stages. The testing dataset results using confusion matrix are shown in Fig 5. Table 4 shows the overall evaluation metrics of testing performance among different EEG channels.

**Table 4:** Testing set performance evaluation among all EEG channels.

Channel	Accuracy %	Sensitivity %	Specificity %	Precision %	F1-Score %	AUC
**C3-A2**	99.11	99.04	99.76	99.01	99.07	0.994
**C4-A1**	99.39	99.34	99.84	99.38	99.36	0.996
**F3-A2**	98.94	98.91	99.73	98.93	98.92	0.993
**F4-A1**	99.35	99.29	99.82	99.36	99.33	0.996
**O1-A2**	98.87	98.83	99.70	98.83	98.83	0.993
**O2-A1**	98.72	98.67	99.67	98.68	98.67	0.992

To comprehensively compare all used channels in the results, the performance among all channels must be calculated and shown for every class. This can be used to determine the best channel for each class because sometimes the overall accuracy of the channel can be affected by some of the classes’ low performance and vice versa. Table 5 shows the detailed performance metrics for each class among all channels using the proposed methodology.

**Table 5:** Testing set performance metrics for each class among all used EEG channels.

Channel	Stage	Accuracy %	Sensitivity %	Specificity %	Precision %	F1-Score %	AUC
**C3-A2**	**N1**	99.22	99.22	99.86	99.04	99.13	0.995
	**N2**	99.32	99.32	99.59	99.10	99.21	0.995
	**N3**	99.00	99.00	99.8	99.19	99.10	0.994
	**R**	98.46	98.46	99.86	99.05	98.76	0.992
	**W**	99.23	99.23	99.74	99.13	99.18	0.995
**C4-A1**	**N1**	99.34	99.34	99.88	99.16	99.25	0.996
	**N2**	99.54	99.54	99.72	99.39	99.47	0.996
	**N3**	99.58	99.58	99.88	99.50	99.54	0.997
	**R**	99.00	99.00	99.93	99.52	99.26	0.995
	**W**	99.26	99.26	99.80	99.33	99.29	0.995
**F3-A2**	**N1**	98.74	98.74	99.80	98.62	98.68	0.993
	**N2**	99.12	99.12	99.51	98.93	99.03	0.993
	**N3**	99.15	99.15	99.74	98.96	99.06	0.995
	**R**	98.94	98.94	99.88	99.17	99.05	0.994
	**W**	98.62	98.62	99.70	98.99	98.80	0.992
**F4-A1**	**N1**	99.40	99.40	99.89	99.22	99.31	0.996
	**N2**	99.49	99.49	99.68	99.30	99.39	0.996
	**N3**	99.42	99.42	99.84	99.35	99.39	0.996
	**R**	98.82	98.82	99.95	99.64	99.23	0.994
	**W**	99.36	99.36	99.80	99.33	99.34	0.996
**O1-A2**	**N1**	98.86	98.86	99.79	98.56	98.71	0.993
	**N2**	99.15	99.15	99.47	98.86	99.00	0.993
	**N3**	98.92	98.92	99.69	98.77	98.85	0.993
	**R**	98.64	98.64	99.81	98.7	98.67	0.992
	**W**	98.59	98.59	99.78	99.26	98.92	0.992
**O2-A1**	**N1**	98.44	98.44	99.82	98.74	98.59	0.991
	**N2**	98.91	98.91	99.49	98.88	98.89	0.992
	**N3**	98.89	98.89	99.67	98.7	98.79	0.993
	**R**	98.52	98.52	99.76	98.41	98.46	0.991
	**W**	98.59	98.59	99.61	98.69	98.64	0.991

Finally, to check and evaluate the robustness of the model performance, it has been evaluated using 10-fold cross-validation, where 10-fold cross-validation is more robust than training-validation-testing methods especially in large datasets. Table 6 shows the 10-fold performance evaluation metrics among different EEG channels, while Table 7 shows the performance of all classes among each channel.

**Table 6:** 10-fold cross-validation performance evaluation among all EEG channels.

Channel	Accuracy %	Sensitivity %	Specificity %	Precision %	F1-Score %	AUC
**C3-A2**	99.05 ± 0.54	98.99 ± 0.58	99.69 ± 0.18	98.95 ± 0.6	99 ± 0.57	0.926 ± 0.04
**C4-A1**	99.32 ± 0.39	99.28 ± 0.41	99.78 ± 0.13	99.31 ± 0.39	99.29 ± 0.41	0.93 ± 0.04
**F3-A2**	98.87 ± 0.65	98.85 ± 0.66	99.66 ± 0.19	98.86 ± 0.65	98.87 ± 0.65	0.925 ± 0.04
**F4-A1**	99.29 ± 0.41	99.24 ± 0.43	99.76 ± 0.14	99.3 ± 0.4	99.27 ± 0.42	0.937 ± 0.04
**O1-A2**	98.8 ± 0.69	98.78 ± 0.7	99.65 ± 0.2	98.78 ± 0.7	98.77 ± 0.7	0.928 ± 0.04
**O2-A1**	98.65 ± 0.77	98.62 ± 0.79	99.61 ± 0.22	98.61 ± 0.79	98.6 ± 0.8	0.939 ± 0.03

**Table 7:** 10-fold cross-validation performance metrics for each class among all used EEG channels.

Channel	Stage	Accuracy %	Sensitivity %	Specificity %	Precision %	F1-Score %	AUC
**C3-A2**	**N1**	99.17 ± 0.56	99.17 ± 0.57	99.8 ± 0.13	98.98 ± 0.69	99.06 ± 0.64	0.93 ± 0.05
	**N2**	99.25 ± 0.51	99.27 ± 0.5	99.54 ± 0.31	99.05 ± 0.65	99.15 ± 0.57	0.93 ± 0.05
	**N3**	98.94 ± 0.72	98.94 ± 0.72	99.74 ± 0.18	99.12 ± 0.59	99.04 ± 0.65	0.94 ± 0.04
	**R**	98.4 ± 1.09	98.39 ± 1.09	99.8 ± 0.14	99 ± 0.68	98.7 ± 0.88	0.92 ± 0.05
	**W**	99.16 ± 0.57	99.17 ± 0.56	99.69 ± 0.21	99.07 ± 0.63	99.13 ± 0.59	0.92 ± 0.05
**C4-A1**	**N1**	99.27 ± 0.5	99.27 ± 0.5	99.82 ± 0.12	99.1 ± 0.61	99.2 ± 0.54	0.93 ± 0.05
	**N2**	99.48 ± 0.35	99.47 ± 0.36	99.65 ± 0.24	99.33 ± 0.46	99.41 ± 0.4	0.93 ± 0.05
	**N3**	99.52 ± 0.32	99.51 ± 0.33	99.81 ± 0.13	99.43 ± 0.39	99.48 ± 0.35	0.93 ± 0.05
	**R**	98.93 ± 0.72	98.95 ± 0.71	99.86 ± 0.09	99.46 ± 0.37	99.21 ± 0.54	0.92 ± 0.05
	**W**	99.2 ± 0.54	99.21 ± 0.54	99.73 ± 0.18	99.26 ± 0.5	99.22 ± 0.53	0.93 ± 0.05
**F3-A2**	**N1**	98.68 ± 0.89	98.68 ± 0.89	99.74 ± 0.17	98.57 ± 0.97	98.62 ± 0.94	0.93 ± 0.05
	**N2**	99.06 ± 0.64	99.06 ± 0.64	99.44 ± 0.38	98.86 ± 0.77	98.97 ± 0.7	0.92 ± 0.05
	**N3**	99.08 ± 0.62	99.08 ± 0.63	99.67 ± 0.23	98.9 ± 0.75	98.99 ± 0.69	0.93 ± 0.05
	**R**	98.89 ± 0.75	98.87 ± 0.76	99.81 ± 0.13	99.1 ± 0.61	99 ± 0.68	0.94 ± 0.04
	**W**	98.57 ± 0.97	98.56 ± 0.97	99.64 ± 0.24	98.94 ± 0.72	98.74 ± 0.85	0.93 ± 0.04
**F4-A1**	**N1**	99.35 ± 0.44	99.34 ± 0.45	99.82 ± 0.12	99.16 ± 0.57	99.24 ± 0.52	0.94 ± 0.04
	**N2**	99.42 ± 0.39	99.42 ± 0.4	99.62 ± 0.26	99.23 ± 0.52	99.34 ± 0.45	0.93 ± 0.05
	**N3**	99.36 ± 0.44	99.36 ± 0.44	99.78 ± 0.15	99.28 ± 0.49	99.33 ± 0.46	0.92 ± 0.05
	**R**	98.77 ± 0.84	98.77 ± 0.83	99.88 ± 0.08	99.58 ± 0.28	99.17 ± 0.56	0.94 ± 0.04
	**W**	99.31 ± 0.47	99.31 ± 0.47	99.74 ± 0.18	99.26 ± 0.5	99.28 ± 0.49	0.93 ± 0.05
**O1-A2**	**N1**	98.81 ± 0.81	98.8 ± 0.82	99.72 ± 0.19	98.49 ± 1.03	98.65 ± 0.92	0.93 ± 0.05
	**N2**	99.08 ± 0.63	99.08 ± 0.62	99.42 ± 0.4	98.79 ± 0.82	98.93 ± 0.73	0.92 ± 0.05
	**N3**	98.85 ± 0.78	98.86 ± 0.77	99.63 ± 0.25	98.71 ± 0.88	98.78 ± 0.83	0.93 ± 0.05
	**R**	98.58 ± 0.96	98.59 ± 0.96	99.74 ± 0.18	98.65 ± 0.92	98.6 ± 0.95	0.94 ± 0.04
	**W**	98.52 ± 1	98.53 ± 1	99.73 ± 0.18	99.2 ± 0.54	98.85 ± 0.78	0.93 ± 0.05
**O2-A1**	**N1**	98.37 ± 1.1	98.38 ± 1.1	99.76 ± 0.17	98.68 ± 0.9	98.53 ± 1	0.93 ± 0.04
	**N2**	98.85 ± 0.78	98.84 ± 0.79	99.43 ± 0.39	98.81 ± 0.81	98.83 ± 0.79	0.94 ± 0.04
	**N3**	98.82 ± 0.8	98.84 ± 0.79	99.6 ± 0.27	98.65 ± 0.92	98.73 ± 0.86	0.94 ± 0.04
	**R**	98.45 ± 1.05	98.47 ± 1.04	99.7 ± 0.21	98.34 ± 1.13	98.39 ± 1.09	0.93 ± 0.05
	**W**	98.52 ± 1.01	98.53 ± 0.99	99.55 ± 0.31	98.64 ± 0.92	98.59 ± 0.96	0.92 0.05±

Strikingly, the results obtained through 10-fold cross-validation closely mirrored those achieved when testing the model on an independent testing set. This convergence underscores the reliability and generalizability of our model, as it consistently demonstrated consistent performance across diverse data subsets. The similarity between cross-validation and testing set results suggests that the model has effectively learned the underlying patterns in the data and can make accurate predictions on unseen instances. This alignment between the two evaluation approaches instills confidence in the model’s reliability and its potential to perform well in real-world scenarios beyond the training data.

## Discussion

The principal objective of this study was to develop a methodology that can classify pattern alterations in the EEG signal due to sleep stages in order to detect at which stage the patient is. This goal has been achieved by creating a deep learning model using CNN that automatically learns how to extract and use the deep features of EEG signal CWT image and discriminate between sleep sages accordingly. After making a comparison between channels’ overall performance in the used methodology, it has been noticed that the best channel among all used channels was the C4-A1 channel; the channel scored performance metrics values of 99.39%, 99.34%, 99.84%, 99.38%, 99.36%, and 0.994 for accuracy, sensitivity, specificity, precision, F1-Score, and Area Under Curve (AUC), respectively. These values can confirm that sleep stage classification can be done using only one EEG channel. The second channel in order in terms of performance was the F4-A1 channel; the channel scored performance metrics values of 99.35%, 99.29%, 99.82%, 99.36%, 99.33%, and 0.996 for accuracy, sensitivity, specificity, precision, F1-Score, and AUC, respectively.

Comparing these two channels together, it can be found that their performances were comparable, and had a small yet significant difference from the third channel in order (C3-A2). However, all other channels scored more than 98.50% in all metrics, which indicated that they were robust as well.

Results in Table 4 showed the best channel that produced the highest accuracy and sensitivity in each class. For class N1, for example, it can be noticed that the best channel for accurately detecting this stage was the F4-A1 channel, with a value of 99.40% for accuracy and 99.40% for sensitivity. For the N2 sleep stage, the best channel was the C4-A1 channel with a value of 99.54% for accuracy and 99.54% for sensitivity. For the N3 sleep stage, the best channel was also the C4-A1 channel with a value of 99.58% for accuracy and 99.58% for sensitivity. For the R sleep stage, the best channel was the C4-A1 channel with a value of 99.00% for accuracy and 90.00% for sensitivity. Finally, for the W stage, the best channel was the F4-A1 channel with a value of 99.36% for accuracy and 99.36% for sensitivity.

Using these results, it has been shown that the C4-A1 channel beat the F4-A1 channel in 3 classes out of 5 classes in terms of performance, making it more suitable for the main classification channel. As a final statement for all shown and discussed results above, it has been noticed that the best EEG channel that can be used as a single input for the CNN model was the C4-A1 channel, either using overall performance or using a single class performance.

The proposed methodology has proved its ability to accurately classify the sleep stages using only one EEG channel by calculating the CWT of each epoch and feeding it to the CNN model. The proposed CNN model is light (19 layers), reflecting its classification speed. Moreover, the proposed methodology has outperformed the methods in literature including those used 1D or 2D CNN models. Table 5 compares the proposed approach to the models and methods that have the highest performance in literature.

For instance, the work proposed by Gupta and Pachori [12] used CWT with Morse, Bump, and Amor mother wavelets images of epochs with transfer learning of pre-trained CNN model called SqueezeNet. So, the authors did not propose any new model to classify CWT images. Moreover, they used a small dataset (42 subjects and 61 PSG) compared to the number used in the current study. Finally, their best overall accuracy was 83.61% using 30-second epochs with Bump mother wavelet and ignoring the results of 150-second epochs because AASM standards recommend using the epoch’s length of only 30 seconds for sleep stage classification.

When comparing the proposed methodology with the closest 2D CNN model performance proposed by Cui et al. [24], their model performance was low because it combined signals to generate 2D images. Such technique was not efficient because time-frequency analysis or representation was used to enhance the difference among different epochs of different sleep stages. The proposed model outperformed that model since the CWT multiresolution time-frequency analysis has been used to make the CNN model capable of extracting very deep features. While the work by Satapathy et al. [14] focused on using pretrained models which are usually not very efficient in EEG sleep stage classification, they also used Wanger-Ville Distribution, which is time-consuming compared to CWT.

On the other hand, Zhu et al. [20] proposed a 1D CNN model composed of 34 layers compared to 19 layers in the current study. Their model used a combination of two EEG channels as input instead of one as in the proposed model. Finally, they used a very small dataset size from only 10 subjects with a total of 12,000 epochs for each channel, which is very small compared to the dataset used in this work that consisted of 100 subjects with a total of 87,001 epochs per channel. Based on that, their model’s performance could vary if they trained it using a larger heterogeneous dataset.

Based on the aforementioned discussion, the model has many merits including: (i) Computational Efficiency, where the 19-layer CNN architecture is designed to be more computationally efficient compared to similar pretrained networks like Densnet (201 layers), ResNet (101 layers), and GoogleNet (144 layers). (ii) Training Speed, with fewer layers, the model will converge faster during the training process. (iii) Resource Efficiency, where the reduced model complexity translates to lower memory requirements during both training and inference, making it more resource-efficient, especially in environments with limited computing resources. (iv) Simpler Architecture, where the 19-layer architecture simplifies the model structure, potentially making it easier to interpret and understand. This simplicity can be advantageous for model debugging, optimization, and maintenance. (v) Reduced Overfitting Risk, because a simpler model is less prone to overfitting, especially when dealing with limited amounts of data. This can contribute to better generalization performance on unseen data.

On the other hand, the proposed model suffers from some limitations such as it may have a limited capacity to capture intricate patterns and representations in complex datasets, its complex architecture may limit the model’s ability to automatically learn hierarchical and abstract features from raw input data, especially when dealing with datasets that have nuanced patterns, in addition, it may have challenges with transfer learning, because the use of pretrained networks for transfer learning might be less straightforward with a significantly different architecture. However, it is very important to note that the mentioned advantages and disadvantages are context-dependent, and the suitability of the 19-layer CNN architecture depends on the specific requirements and constraints of the EEG classification task at hand.

Moving forward, efforts to enhance the interpretability of the deep learning model and address uncertainties in single-channel classification could focus on key areas. Incorporating interpretable deep learning techniques, such as layer-wise relevance propagation and attention mechanisms will offer insights into influential features within the EEG signal [38–40]. Developing specific metrics for model explainability and employing visualizations such as heatmaps can provide tangible representations of the model’s decision-making process, aiding clinician understanding and trust. Additionally, uncertainty estimation techniques, ensemble models, and robustness analysis can be explored to quantify confidence, improve reliability, and assess the model’s generalization to variations [41].

In parallel, tailored methods for explaining decisions with single-channel inputs, interactive interfaces for clinician collaboration, and feedback mechanisms for continuous improvement can further refine the diagnostic process. Integration with existing sleep studies or complementary modalities, such as polysomnography, presents an avenue for a more comprehensive understanding of sleep patterns [39, 41]. These future directions collectively aim to advance the explainability and reliability of the deep learning model in the context of EEG-based sleep stage classification [38, 40].

Finally, as a summary of comparing the proposed methodology with surveyed literature, the proposed methodology has outperformed most previous works either in terms of dataset size or in terms of the performance of the classifier. Moreover, the proposed CNN model is lighter than all models in literature so that it is feasible to be used as a time sleep stages classifier. Table 8 shows a comparison in terms of accuracy between literature and proposed method.

**Table 8:** Comparing the accuracy of the proposed methodology to literature.

Reference	Methodology	Accuracy %	
[2]	Time, frequency, and time-frequency features with SVM	94.58	
		92.04	
[26]	1D-CNN	97.22	
[27]	Attention-based 1D-CNN with two datasets	93.7	
		82.8	
[31]	2D CNN model	91.2	
[11]	Squeezenet model with the continuous wavelet transform with 30 seconds	Morse	83.34
		Bump	83.61
		Amor	83.17
[12]	Time domain and frequency domain features with SVM	92	
[29]	1D-CNN	2 Classes	98.06
		3 classes	94.64
		4 classes	92.36
		5 classes	91.22
		6 classes	91.00
Proposed	2D-CNN with CWT	C3-A2	99.11
		C4-A1	99.39
		F3-A2	98.94
		F4-A1	98.35
		O1-A2	98.87
		O2-A1	98.71

## Conclusions

In conclusion, a new methodology for sleep stage classification using EEG channels has been proposed focusing on high accuracy. The proposed system developed a new algorithm for detecting and classifying sleep stages by providing a new light CNN model for classification purposes. The proposed methodology can produce a precise and accurate sleep stage classification by converting each 30-second epoch into images using CWT. In addition, the effectiveness of the proposed system was evaluated using different performance evaluation metrics, which showed that the proposed methodology was robust and highly accurate. Finally, the best channel that can be used as input to the methodology was C4-A1 followed by F4-A1. The proposed system may be used with patients suffering from stroke. Other applications may include the study of sleep quality or monitoring brain signals during sleeping time.
